# Association of moderate alcohol intake with in vivo amyloid-beta deposition in human brain: A cross-sectional study

**DOI:** 10.1371/journal.pmed.1003022

**Published:** 2020-02-25

**Authors:** Jee Wook Kim, Min Soo Byun, Dahyun Yi, Jun Ho Lee, Kang Ko, So Yeon Jeon, Bo Kyung Sohn, Jun-Young Lee, Yu Kyeong Kim, Seong A Shin, Chul-Ho Sohn, Dong Young Lee, for the KBASE Research Group

**Affiliations:** 1 Department of Neuropsychiatry, Hallym University Dongtan Sacred Heart Hospital, Hwaseong, Republic of Korea; 2 Department of Psychiatry, Hallym University College of Medicine, Chuncheon, Republic of Korea; 3 Institute of Human Behavioral Medicine, Medical Research Center, Seoul National University, Seoul, Republic of Korea; 4 Department of Neuropsychiatry, Seoul National University Hospital, Seoul, Republic of Korea; 5 Department of Geriatric Psychiatry, National Center for Mental Health, Seoul, Republic of Korea; 6 Department of Psychiatry, Chungnam National University Hospital, Daejeon, Republic of Korea; 7 Department of Psychiatry, Sanggye Paik Hospital, Inje University College of Medicine, Seoul, Republic of Korea; 8 Department of Neuropsychiatry, SMG-SNU Boramae Medical Center, Seoul, Republic of Korea; 9 Department of Psychiatry, Seoul National University College of Medicine, Seoul, Republic of Korea; 10 Department of Nuclear Medicine, SMG-SNU Boramae Medical Center, Seoul, Republic of Korea; 11 Department of Radiology, Seoul National University College of Medicine, Seoul, Republic of Korea; Columbia University, UNITED STATES

## Abstract

**Background:**

An emerging body of literature has indicated that moderate alcohol intake may be protective against Alzheimer disease (AD) dementia. However, little information is available regarding whether moderate alcohol intake is related to reductions in amyloid-beta (Aβ) deposition, or is protective via amyloid-independent mechanisms in the living human brain. Here we examined the associations of moderate alcohol intake with in vivo AD pathologies, including cerebral Aβ deposition, neurodegeneration of AD-signature regions, and cerebral white matter hyperintensities (WMHs) in the living human brain.

**Methods and findings:**

The present study was part of the Korean Brain Aging Study for Early Diagnosis and Prediction of Alzheimer’s Disease (KBASE), an ongoing prospective cohort study that started in 2014. As of November 2016, 414 community-dwelling individuals with neither dementia nor alcohol-related disorders (280 cognitively normal [CN] individuals and 134 individuals with mild cognitive impairment [MCI]) between 56 and 90 years of age (mean age 70.9 years ± standard deviation 7.8; male, *n* [%] = 180 [43.5]) were recruited from 4 sites (i.e., 2 university hospitals and 2 public centers for dementia prevention and management) around Seoul, South Korea. All the participants underwent comprehensive clinical assessments comprising lifetime and current histories of alcohol intake and multimodal brain imaging, including [^11^C] Pittsburgh compound B positron emission tomography (PET), [^18^F] fluorodeoxyglucose (FDG) PET, and magnetic resonance imaging (MRI) scans. Lifetime and current alcohol intake were categorized as follows: no drinking, <1 standard drink (SD)/week, 1–13 SDs/week, and 14+ SDs/week. A moderate lifetime alcohol intake (1–13 SDs/week) was significantly associated with a lower Aβ positivity rate compared to the no drinking group, even after controlling for potential confounders (odds ratio 0.341, 95% confidence interval 0.163–0.714, *p* = 0.004). In contrast, current alcohol intake was not associated with amyloid deposition. Additionally, alcohol intake was not related to neurodegeneration of AD-signature regions or cerebral WMH volume. The present study had some limitations in that it had a cross-sectional design and depended on retrospective recall for alcohol drinking history.

**Conclusions:**

In this study, we observed in middle- and old-aged individuals with neither dementia nor alcohol-related disorders that moderate lifetime alcohol intake was associated with lower cerebral Aβ deposition compared to a lifetime history of not drinking. Moderate lifetime alcohol intake may have a beneficial influence on AD by reducing pathological amyloid deposition rather than amyloid-independent neurodegeneration or cerebrovascular injury.

## Abstract 2

In a cross-sectional study, Jee Wook Kim and colleagues investigate the association of moderate alcohol intake with in vivo amyloid-beta deposition in human brain.

## Author summary

**Why was this study done?:**

- An emerging body of literature has indicated that moderate alcohol intake may be protective against Alzheimer disease (AD) dementia.
- Preclinical studies from animal and cell culture models have demonstrated that moderate alcohol intake confers protection by attenuating molecular amyloid-beta (Aβ) pathology.
- Little information is available regarding whether moderate alcohol intake is related to decreased Aβ deposition, or is protective via amyloid-independent mechanisms in the living human brain.

**What did the researchers do and find?:**

- We performed cross-sectional analyses of the baseline data from 414 middle- and old-aged individuals with neither dementia nor alcohol-related disorders who participated in the Korean Brain Aging Study for Early Diagnosis and Prediction of Alzheimer’s Disease (KBASE), an ongoing prospective cohort study.
- All participants were systematically interviewed on current and past alcohol intake and underwent brain imaging to examine the associations of alcohol intake with various in vivo AD pathologies.
- We observed that moderate lifetime alcohol intake (i.e., 1–13 standard drinks [SDs]/week) was significantly associated with lower amyloid deposition compared to no drinking, whereas current alcohol intake did not affect amyloid deposition.
- In contrast, neither lifetime nor current alcohol intake was associated with neurodegeneration of the AD–signature cortical regions or cerebral white matter injury.

**What do these findings mean?:**

- The present findings from middle- and old-aged individuals with neither dementia nor alcohol-related disorders suggest that moderate lifetime alcohol intake may have a beneficial influence on AD by reducing pathological amyloid deposition.
- The findings should be cautiously interpreted because the present study had a cross-sectional design and depended on retrospective recall for alcohol drinking history.

## Introduction

Debate remains regarding whether alcohol intake has protective or harmful effects with respect to the risk of dementia or cognitive decline. Although excessive alcohol intake is associated with an increased risk of dementia or cognitive decline [1–4], an emerging body of literature has indicated that moderate alcohol intake may be protective against both conditions [3,5–16].

Several human studies have reported that moderate alcohol intake is associated with a lower risk of Alzheimer disease (AD) dementia [3,9–13]. Neurobiological findings from animal and cell culture models have demonstrated that moderate alcohol intake confers protection by attenuating molecular amyloid-beta (Aβ) pathology and blocking Aβ-induced damage [17–20]. Moderate alcohol intake decreased Aβ peptides in AD transgenic mice by promoting the non-amyloidogenic processing of amyloid precursor protein [17]. Moderate concentrations of alcohol also protected cultured hippocampal neurons against Aβ-induced neurotoxicity [18,19]. Furthermore, pretreatment with a moderate level of alcohol reduced Aβ aggregation and prevented soluble Aβ toxicity in cultured cells by blocking the formation of stable Aβ dimers with high cellular toxicity [20]. However, little information is available regarding whether moderate alcohol intake is related to decreased Aβ deposition in the human brain in vivo.

Previous findings for the influence of moderate alcohol intake on neurodegeneration and cerebrovascular changes are controversial [21–30]. While many preclinical studies and some human magnetic resonance imaging (MRI) studies suggested that moderate alcohol intake has a protective effect against neurodegenerative or cerebrovascular injury [21–28], others did not observe a protective effect of alcohol [29,30]. Although a postmortem pathological study did not demonstrate any association between alcohol consumption and neuropathological lesions, the study relied on only semi-quantitative assessment for AD pathologies restricted to only a few brain regions [31].

We aimed to test the hypothesis that moderate alcohol intake is associated with reduced cerebral Aβ deposition in middle- and old-aged individuals with neither dementia nor alcohol-related disorders [32]. Cerebral Aβ deposition was measured by [^11^C] Pittsburg compound B (PiB) positron emission tomography (PET). We also tested the hypothetical associations of moderate alcohol intake with AD-related neurodegeneration and cerebrovascular white matter injury. The neurodegeneration of AD-signature regions was measured by both MRI and [^18^F] fluorodeoxyglucose (FDG) PET imaging. Cerebral white matter hyperintensities (WMHs) on MRI were used as a measure of cerebrovascular injury [33,34]. Additionally, we explored the associations between other categories of alcohol intake and the neuroimaging biomarkers mentioned above.

## Methods

### Participants

The present study was part of the Korean Brain Aging Study for Early Diagnosis and Prediction of Alzheimer’s Disease (KBASE), an ongoing prospective cohort study that started in 2014. The KBASE study aimed to search for new AD biomarkers and investigate how multifaceted lifetime experiences and bodily changes contribute to the brain changes related to AD. The current study analyzed the data of 414 individuals without dementia (280 cognitively normal [CN] individuals and 134 individuals with mild cognitive impairment [MCI]) between 56 and 90 years of age (mean age 70.9 ± standard deviation 7.8; male, *n* [%] = 180 [43.5]) who were recruited as of 30 November 2016. Participants were recruited through 4 recruitment sites around Seoul, South Korea. Potentially eligible individuals who participated in a dementia screening program at 2 public centers for dementia prevention and management or visited memory clinics at 2 university hospitals (i.e., Seoul National University Hospital and SNU-SMG Boramae Medical Center) around Seoul, South Korea, were informed about study participation, and those who volunteered were invited for an assessment of eligibility. In addition, volunteers from the community were recruited through advertisements online, posters and brochures at the main recruitment sites, and word of mouth (recommended by other participants, family members, friends, or acquaintances). More detailed information on KBASE study characteristics including recruitment has been published previously [35]. The CN group consisted of participants with a Clinical Dementia Rating (CDR) [36] score of 0 and no diagnosis of MCI or dementia. All individuals with MCI met the current consensus criteria for amnestic MCI, which are as follows: (1) memory complaints confirmed by an informant, (2) objective memory impairments, (3) preserved global cognitive function, (4) independence in functional activities, and (5) no dementia. Regarding criterion 2, the age-, education-, and sex-adjusted *z*-scores for at least 1 of 4 episodic memory tests was <−1.0. The 4 memory tests were the Word List Memory, Word List Recall, Word List Recognition, and Constructional Recall tests, which are included in the Korean version of the Consortium to Establish a Registry for Alzheimer’s Disease (CERAD-K) neuropsychological assessment battery [37]. All individuals with MCI had a CDR score of 0.5.

The exclusion criteria were as follows: (1) presence of a major psychiatric illness, including alcohol-related disorders; (2) significant neurological or medical conditions or comorbidities that could affect mental function; (3) contraindications for an MRI scan (e.g., pacemaker or claustrophobia); (4) illiteracy; (5) the presence of significant visual/hearing difficulties and/or severe communication or behavioral problems that would make clinical examinations or brain scans difficult; (6) taking an investigational drug; and (7) pregnant or breastfeeding.

The study protocol was approved by the institutional review boards of Seoul National University Hospital (C-1401-027-547) and SNU-SMG Boramae Medical Center (26-2015-60), Seoul, South Korea, and the study was conducted in accordance with the recommendations of the current version of the Declaration of Helsinki. The participants or their legal representatives gave written informed consent.

### Clinical assessments

All participants underwent comprehensive clinical and neuropsychological assessments administered by trained psychiatrists and neuropsychologists based on the KBASE assessment protocol [35], which incorporates the CERAD-K neuropsychological assessment battery [38,39].

### Assessment of alcohol intake

All participants were systematically interviewed by trained nurses to determine alcohol intake using an interview form (S1 Interview Form) at the medical science building in the Seoul National University medical campus, Seoul, South Korea. According to the World Health Organization guideline [40], 1 standard drink (SD) was defined as any drink that contained 10 grams of pure alcohol. Because there are a wide variety of alcoholic beverages and brands, different beverages were categorized as follows: 1 can of beer (4.5% alcohol; 330 ml) = 1 SD; 1 bottle of beer (4.5% alcohol; 640 ml) = 2 SDs, 1 bottle of local Korean spirit (20% alcohol; 360 ml) = 6 SDs, 1 bottle of spirit (40% alcohol; 750 ml) = 24 SDs, 1 bottle of Korean traditional wine (8% alcohol; 900 ml) = 6 SDs, and 1 bottle of wine (12% alcohol; 900 ml) = 9 SDs. Additionally, the patterns of alcohol intake for each participant were categorized as follows: drinking status (non-drinker, former drinker, drinker) and frequency and amount of alcohol intake (SDs/drinking day, drinking days/week, SDs/week) during the past year (current) and lifetime. Among drinkers, those who drank more than 6 SDs per drinking day in the past year were sub-classified as binge drinkers [10]. Among current non-drinkers, those who used to drink regularly but have not drunk alcohol in the past year were sub-classified as former drinkers [5,11]. Previous epidemiological studies on the effect of alcohol intake showed that there was a clear difference in the risk of overall or AD dementia among no drinking (reference), former drinking, mild drinking, moderate drinking, and high drinking groups [11,22,41]. Based on these reports, our participants were categorized based on weekly alcohol intake as follows: no drinking (reference category), former drinking, <1 SD/week (<10 grams/week; mild drinking), 1–13 SDs/week (10–130 grams/week; moderate drinking), and 14+ SDs/week (≥140 grams/week; unsafe drinking in the newly revised UK Department of Health guidelines [42]).

### Assessment of potential confounders

Alcohol intake may be influenced by various other conditions. Therefore, all participants were systematically evaluated about potential confounders, such as depression, vascular risk, body weight, body mass index (BMI), occupational complexity, annual income, and apolipoprotein E (APOE) genotyping. The Geriatric Depression Scale (GDS) [43] was used to assess depression. Body weight, height, and comorbidity rates of vascular risk factors (including hypertension, diabetes mellitus, dyslipidemia, coronary heart disease, transient ischemic attack, and stroke) were assessed based on data collected by trained nurses during systematic interviews of participants and their informants; BMI was calculated as weight in kilograms divided by the square of height in meters, and a vascular risk score was calculated based on the number of vascular risk factors [44]. With regard to occupational complexity, we considered only the longest-held occupation, classified into 4 levels based on the skill levels described in International Standard Classification of Occupations [45]. Occupations at skill level 1 typically involve simple and routine physical or manual tasks. Occupations at skill level 2 include the performance of tasks such as operating machinery and electronic equipment, driving vehicles, maintenance and repair of electrical and mechanical equipment, and manipulation, ordering, and storage of information. Occupations at skill level 3 include the performance of complex technical and practical tasks that require complex problem-solving, reasoning, and decision-making in a specialized field. Occupations at skill level 4 involve the performance of tasks that require complex problem-solving, decision-making, and creativity based on an extensive body of theoretical and factual knowledge in a specialized field. Information about occupation was obtained from self-report by the participants and confirmed by reliable informants. Annual income was evaluated and categorized into 3 groups (below the minimum cost of living [MCL], at or more than the MCL but below twice the MCL, twice the MCL or more; http://www.law.go.kr). The MCL was determined according to the administrative rule published by the Ministry of Health and Welfare, Republic of Korea, in November 2012. The MCL was 572,168 Korean Won (KRW) (equivalent to US$507.9) for a single-person household and added 286,840 KRW (equivalent to US$254.6) for each additional household member. Blood samples were obtained via venipuncture, genomic DNA was extracted from whole blood, and APOE genotyping was performed as previously described [46]. apolipoprotein ε4 (APOE4) positivity was coded if at least 1 ε4 allele was present.

### Measurement of cerebral Aβ deposition

All participants underwent simultaneous 3D PiB PET and 3D T1-weighted MRI scans using a 3.0T Biograph mMR (PET-MR) scanner (Siemens) according to the manufacturer’s guidelines; the details of PiB PET imaging acquisition and preprocessing are provided in S1 Method. The automatic anatomic labeling algorithm and a region-combining method [47] were applied to determine regions of interest (ROIs), to characterize the PiB retention levels in the frontal, lateral parietal, posterior cingulate/precuneus, and lateral temporal regions. The standardized uptake value ratio (SUVR) values for each ROI were calculated by dividing the mean value for all voxels within each ROI by the mean cerebellar uptake value in the same image. A global cortical ROI consisting of 4 ROIs was also defined, and a global Aβ retention value was generated by dividing the mean value for all voxels of the global cortical ROI by the mean cerebellar uptake value in the same image [47,48]. Participants were classified as Aβ+ if global Aβ retention was >1.21, and as Aβ− if global Aβ retention was ≤1.21 [49].

### Measurement of AD-signature neurodegeneration

All participants underwent FDG PET imaging using the abovementioned PET-MR scanner; the details of FDG PET image acquisition and preprocessing are provided in S1 Method. AD-signature FDG ROIs, such as the angular gyri, posterior cingulate cortex, and inferior temporal gyri, which are sensitive to the changes associated with AD [50], were determined. AD-signature cerebral glucose metabolism (AD-CM) was defined as the voxel-weighted mean SUVR extracted from the AD-signature FDG ROIs; the details of MRI acquisition and preprocessing are provided in S1 Method. AD-signature cortical thickness (AD-CT) was defined as the mean cortical thickness values obtained from AD-signature regions including the entorhinal, inferior temporal, middle temporal, and fusiform gyrus regions, as previously described [50].

### Measurement of WMHs

All participants underwent MRI scans with fluid-attenuated inversion recovery (FLAIR) using the abovementioned 3.0T PET-MR scanner; the details of the volume measurements of cerebral WMHs are provided in S1 Method.

### Statistical analysis

In order to test the hypothetical associations between moderate alcohol intake and neuroimaging biomarkers and to explore the association between other categories of alcohol intake and the biomarkers, we planned to perform multiple regression analyses as follows. First, multiple logistic regression analyses with lifetime (or current) alcohol intake category (i.e., no drinking, <1 SD/week, 1–13 SDs/week, and 14+ SDs/week) as the independent variable and Aβ positivity as the dependent variable were conducted. In these analyses, to compare the effect of alcohol intake relative to no drinking, no drinking was used as the reference (i.e., no drinking versus <1 SD/week, no drinking versus 1–13 SDs/week, or no drinking versus 14+ SDs/week) within each model. Three models were tested, with stepwise control of the potential confounders that could affect the association between alcohol intake and AD biomarkers. The first model (Model 1) did not include any covariates; the second model (Model 2) included age, sex, APOE4, vascular risk score, and GDS score as covariates; and the third model (Model 3) included the covariates in the second model plus education (high school education or below versus more than high school education), clinical diagnosis (CN versus MCI), occupational complexity, annual income, body weight, and BMI [51]. Second, multiple linear regression analyses were performed to compare the differences in global Aβ retention, AD-CM, AD-CT, and WMHs among the lifetime (or current) alcohol intake groups while controlling for the same covariates. In the analyses, global Aβ retention was used after natural log-transformation to achieve normal distribution.

As sensitivity analyses, we also performed the same analyses after excluding binge drinkers from lifetime or current drinkers in order to reduce the influence of the binge drinking pattern on the association between the frequency and amount of alcohol intake and the neuroimaging variables. Additionally, we did the same analyses after excluding former drinkers from current non-drinkers to minimize the potential effects of forced abstainers who stopped using alcohol because of other health concerns related to problem drinking.

In order to investigate the influence of age (younger [<75 years] versus older [≥75 years]) [52], sex (female versus male), APOE4 (APOE4+ versus APOE4−), and clinical diagnosis (CN versus MCI)] on the association between alcohol intake and neuroimaging biomarkers that were significant in the analyses described above, the same regression analysis was repeated including a 2-way interaction term between alcohol intake and each of the 4 neuroimaging biomarkers as an additional independent variable.

All statistical analyses were performed using IBM SPSS Statistics 24.

## Results

### Participant characteristics

The demographic and clinical characteristics of the participants are presented in Tables 1 and S1.

**Table 1:** Demographic and clinical characteristics of participants by category of lifetime alcohol intake.

Characteristic	Lifetime alcohol intake category				*p-*Value
	No drinking	<1 SD/week	1–13 SDs/week	14+ SDs/week	
*N*	227	16	125	46	
Age, years	71.8 (7.3)	70.1 (7.5)	69.6 (8.2)	70.1 (8.8)	0.077^a^
Male, *n/N* (%)	38/227 (16.7)	8/16 (50.0)	91/125 (72.8)	43/46 (93.5)	<0.001^b^
APOE4 positivity, *n/N* (%)	53/226 (23.5)	1/16 (6.3)	29/125 (23.2)	15/46 (32.6)	0.192^b^
CN, *n/N* (%)	142/227 (62.6)	12/16 (75.0)	95/125 (76.0)	31/46 (67.4)	0.070^b^
MMSE	25.2 (3.5)	27.4 (2.8)	25.5 (3.4)	25.7 (3.5)	0.082^a^
Body weight, kg	58.2 (8.9)	62.6 (9.0)	64.7 (1.0)	66.5 (7.0)	<0.001^a^
BMI, kg/m^2^	24.4 (3.1)	24.2 (2.9)	24.3 (3.1)	24.3 (2.6)	0.955^a^
Vascular risk score	1.1 (1.0)	1.1 (0.9)	0.9 (1.0)	1.1 (1.0)	0.480^a^
GDS score	6.6 (5.8)	2.1 (1.9)	6.3 (6.9)	7.3 (6.9)	0.028^a^
Education more than high school, *n/N* (%)	61/227 (26.9)	9/16 (56.3)	56/125 (44.8)	16/46 (34.8)	0.002^b^
Occupational complexity					<0.001^c^
None, *n/N* (%)	65/226 (28.8)	2/16 (12.5)	8/125 (6.4)	0/46 (0.0)	
Skill level 1, *n/N* (%)	16/226 (7.1)	0/16 (0.0)	11/125 (8.8)	1/46 (2.2)	
Skill level 2, *n/N* (%)	71/226 (31.4)	5/16 (31.3)	42/125 (33.6)	19/46 (41.3)	
Skill level 3, *n/N* (%)	24/226 (10.6)	0/16 (0.0)	23/125 (18.4)	8/46 (17.4)	
Skill level 4, *n/N* (%)	50/226 (22.1)	9/16 (56.3)	41/125 (32.8)	18/46 (39.1)	
Annual income status					0.702^b^
<MCL, *n/N* (%)	18/227 (7.9)	0/16 (0.0)	11/125 (8.8)	5/46 (10.9)	
≥MCL, <2×MCL, *n/N* (%)	98/227 (43.2)	8/16 (50.0)	57/125 (45.6)	24/46 (52.2)	
≥2×MCL, *n/N* (%)	111/227 (48.9)	8/16 (50.0)	17/125 (30.0)	193/46 (46.6)	
Cerebral Aβ deposition					
Aβ positivity, *n/N* (%)	79/223 (35.4)	5/16 (31.3)	23/121 (19.0)	12/46 (26.1)	0.015^b^
Global Aβ retention, SUVR	1.32 (0.4)	1.35 (0.5)	1.24 (0.3)	1.25 (0.3)	0.151^a^
Neurodegeneration					
AD-CM, SUVR	1.38 (0.1)	1.40 (0.1)	1.41 (0.1)	1.40 (0.1)	0.347^a^
AD-CT, mm	2.80 (0.2)	2.80 (0.2)	2.82 (0.2)	2.81 (0.3)	0.826^a^
WMH volume, cm^3^	6.15 (5.7)	7.61 (6.6)	5.46 (4.7)	5.87 (5.6)	0.498^a^

Data are expressed as mean (standard deviation) unless otherwise indicated.

^a^By 1-way analysis of variance.

^b^By chi-squared test.

^c^By Fisher’s exact test.

AD-CM, Alzheimer disease–signature cerebral glucose metabolism; AD-CT, Alzheimer disease–signature cortical thickness; APOE4, apolipoprotein β4; Aβ, amyloid-beta; BMI, body mass index; CN, cognitively normal; GDS, Geriatric Depression Scale; MCL, minimum cost of living; MMSE, Mini-Mental State Examination; SD, standard drink; SUVR, standardized uptake value ratio; WMH, white matter hyperintensity.

### Association of alcohol intake with cerebral amyloid deposition

**Fig 1:**
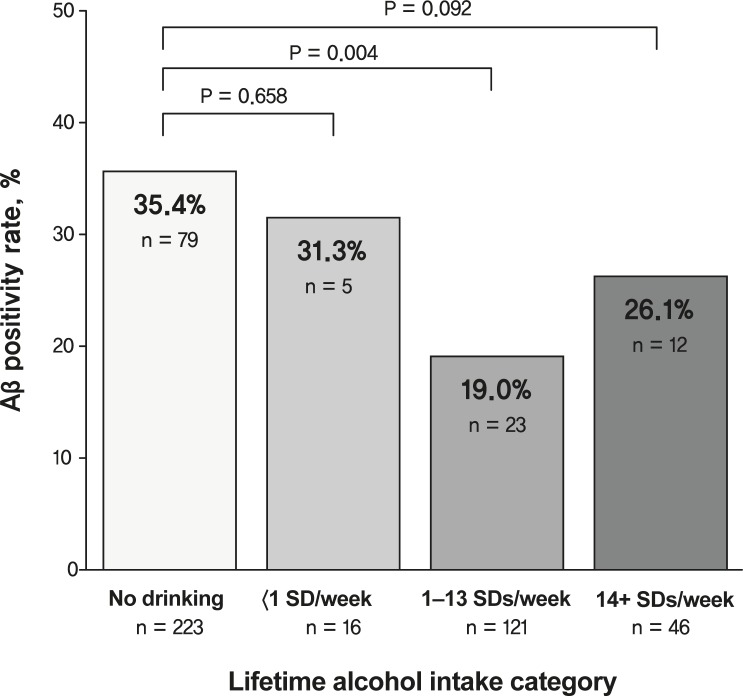
Aβ positivity rate according to lifetime alcohol intake category. Comparison of Aβ positivity rate for no drinking versus <1 SD/week, no drinking versus 1–13 SDs/week, and no drinking versus 14+ SDs/week. Multiple logistic regression analyses were performed after adjusting for age, sex, apolipoprotein ε4, vascular risk score, Geriatric Depression Scale score, education, clinical diagnosis, occupational complexity, annual income, body weight, and body mass index. Aβ, amyloid-beta; SD, standard drink.

The multiple logistic regression analyses revealed that a lifetime alcohol intake of 1–13 SDs/week was significantly associated with lower Aβ positivity compared to the no drinking group, even after controlling for potential confounders (Models 1, 2, and 3), while lifetime alcohol intakes of <1 SD/week and 14+ SDs/week were not related to Aβ positivity (Table 2; Fig 1). The results were similar even when different thresholds for Aβ positivity (i.e., global Aβ retention > 1.19 [49] or >1.40 [50] instead of >1.21) were applied to define the Aβ positive state (S2 Table). Similarly, the multiple regression analyses revealed significant (Models 1 and 2) association between a lifetime alcohol intake of 1–13 SDs/week and global Aβ retention (Table 2). Meanwhile, current alcohol intake was not related to Aβ positivity or global Aβ retention (Table 2).

**Table 2:** Results of the multiple logistic and linear regression analyses assessing the associations of stratified alcohol intake with Aβ deposition in participants overall.

Alcohol intake	Aβ positivity	Aβ retention, SUVR
	OR (95% CI)^†^, *p-*Value	B (95% CI)^‡^, *p-*Value
**Lifetime**		
Model 1^a^		
<1 SD/week	0.850 (0.285 to 2.535), 0.771	0.014 (−0.105 to 0.132), 0.822
1–13 SDs/week	0.439 (0.258 to 0.747), 0.002	−0.057 (−0.108 to −0.005), 0.032
14+ SDs/week	0.660 (0.323 to 1.348), 0.254	−0.046 (−0.121 to 0.028), 0.220
Model 2^b^		
<1 SD/week	1.508 (0.460 to 4.935), 0.498	0.061 (−0.050 to 0.172), 0.279
1–13 SDs/week	0.335 (0.166 to 0.678), 0.002	−0.059 (−0.116 to −0.003), 0.040
14+ SDs/week	0.411 (0.158 to 1.070), 0.069	−0.072 (−0.153 to 0.009), 0.080
Model 3^c^		
<1 SD/week	1.316 (0.390 to 4.437), 0.658	0.038 (−0.066 to 0.142), 0.471
1–13 SDs/week	0.341 (0.163 to 0.714), 0.004	−0.047 (−0.100 to 0.006), 0.084
14+ SDs/week	0.423 (0.156 to 1.150), 0.092	−0.063 (−0.139 to 0.013), 0.103
**Current**		
Model 1^a^		
<1 SD/week	0.872 (0.324 to 2.344), 0.785	0.009 (−0.097 to 0.115), 0.865
1–13 SDs/week	0.486 (0.266 to 0.886), 0.019	−0.060 (−0.117 to −0.002), 0.042
14+ SDs/week	0.488 (0.193 to 1.233), 0.129	−0.046 (−0.133 to 0.040), 0.294
Model 2^b^		
<1 SD/week	1.178 (0.389 to 3.564), 0.772	0.035 (−0.063 to 0.134), 0.485
1–13 SDs/week	0.496 (0.245 to 1.004), 0.051	−0.040 (−0.096 to 0.016), 0.162
14+ SDs/week	0.723 (0.244 to 2.141), 0.558	−0.008 (−0.095 to 0.080), 0.858
Model 3^c^		
<1 SD/week	1.234 (0.398 to 3.824), 0.716	0.031 (−0.061 to 0.122), 0.508
1–13 SDs/week	0.503 (0.241 to 1.052), 0.068	−0.032 (−0.084 to 0.021), 0.235
14+ SDs/week	0.804 (0.261 to 2.477), 0.704	<0.001 (−0.081 to 0.082), 0.995

Global Aβ retention was used after natural log-transformation to achieve normal distribution.

^†^By multiple logistic regression analysis (no drinking served as the reference group).

^‡^By multiple linear regression analysis (no drinking served as the reference group).

^a^Not adjusted.

^b^Adjusted for age, sex, apolipoprotein ε4, vascular risk score, and Geriatric Depression Scale score.

^c^Adjusted for covariates in Model 2 plus education, clinical diagnosis, occupational complexity, annual income, body weight, and body mass index.

Aβ, amyloid-beta; B, unstandardized regression coefficient; OR, odds ratio; SD, standard drink; SUVR, standardized uptake value ratio.

### Association of alcohol intake with neurodegeneration and WMHs

No group differences were observed in AD-CM, AD-CT, or WMHs between the lifetime (or current) alcohol intake categories. The findings for the association between alcohol intake and neurodegeneration or WMHs were not changed even after additional controlling for all the covariates (Table 3).

**Table 3:** Results of the multiple linear regression analyses assessing the associations of stratified alcohol intake with AD-CM, AD-CT, and WMHs in participants overall.

Alcohol intake	B (95% CI)^†^, *p-*Value		
	AD-CM, SUVR	AD-CT, mm	WMHs, cm^3^
**Lifetime**			
Model 1^a^			
<1 SD/week	0.020 (−0.047 to 0.088),0.555	<0.001 (−0.113 to 0.113), 0.998	1.430 (−1.635 to 4.495), 0.359
1–13 SDs/week	0.027 (−0.003 to 0.056), 0.075	0.023 (−0.027 to 0.074), 0.360	−0.715 (−1.985 to 0.556), 0.269
14+ SDs/week	0.016 (−0.026 to 0.059), 0.447	0.013 (−0.058 to 0.085), 0.718	−0.307 (−2.163 to 1.549), 0.745
Model 2^b^			
<1 SD/week	0.013 (−0.055 to 0.080), 0.713	−0.031 (−0.129 to 0.068), 0.543	1.495 (−1.569 to 4.558),0.338
1–13 SDs/week	0.024 (−0.011 to 0.058), 0.173	0.014 (−0.037 to 0.065), 0.593	−1.085 (−2.577 to 0.407), 0.153
14+ SDs/week	0.023 (−0.026 to 0.072), 0.359	0.030 (−0.043 to 0.103), 0.417	−1.071 (−3.225 to 1.084), 0.329
Model 3^c^			
<1 SD/week	0.018 (−0.047 to 0.084), 0.578	−0.017 (−0.110 to 0.076), 0.717	1.663 (−1.429 to 4.755), 0.291
1–13 SDs/week	0.016 (−0.017 to 0.050), 0.343	0.004 (−0.044 to 0.052), 0.873	−1.025 (−2.535 to 0.485), 0.183
14+ SDs/week	0.017 (−0.031 to 0.065, 0.481	0.038 (−0.031 to 0.107), 0.282	−0.909 (−3.099 to 1.281), 0.415
**Current**			
Model 1^a^			
<1 SD/week	0.026 (−0.034 to 0.087), 0.391	0.015 (−0.086 to 0.115), 0.771	0.397 (−2.293 to 3.087), 0.772
1–13 SDs/week	0.015 (−0.018 to 0.048), 0.366	0.033 (−0.023 to 0.088), 0.248	−0.139 (−1.563 to 1.285), 0.848
14+ SDs/week	0.039 (−0.010 to 0.088), 0.115	0.109 (0.026 to 0.191), 0.010	−1.121 (−3.261 to 1.102), 0.304
Model 2^b^			
<1 SD/week	0.023 (−0.037 to 0.082), 0.455	0.001 (−0.086 to 0.088), 0.982	0.513 (−2.144 to 3.69), 0.704
1–13 SDs/week	0.008 (−0.026 to 0.042), 0.655	0.012 (−0.038 to 0.063), 0.626	−0.080 (−1.552 to 1.393), 0.915
14+ SDs/week	0.022 (−0.030 to 0.074), 0.411	0.067 (−0.011 to 0.144), 0.090	−0.925 (−3.202 to 1.353), 0.425
Model 3^c^			
<1 SD/week	0.023 (−0.034 to 0.081), 0.427	−0.001 (−0.082 to 0.081), 0.986	0.473 (−2.198 to 3.144), 0.728
1–13 SDs/week	0.004 (−0.029 to 0.037), 0.818	0.003 (−0.044 to 0.051), 0.894	−0.070 (−1.552 to 1.412), 0.926
14+ SDs/week	0.020 (−0.031 to 0.070), 0.443	0.067 (−0.006 to 0.140), 0.070	−0.920 (−3.210 to 1.371), 0.430

^†^By multiple linear regression analysis (no drinking served as the reference group).

^a^Not adjusted.

^b^Adjusted for age, sex, apolipoprotein ε4, vascular risk score, and Geriatric Depression Scale score.

^c^Adjusted for covariates in Model 2 plus education, clinical diagnosis, occupational complexity, annual income, body weight, and body mass index.

AD-CM, Alzheimer disease–signature cerebral glucose metabolism; AD-CT, Alzheimer disease–signature cortical thickness; B, unstandardized regression coefficient; SD, standard drink; SUVR, standardized uptake value ratio; WMH, white matter hyperintensity.

### Sensitivity analyses

Sensitivity analyses including only participants without binge drinking showed similar results (S3 and S4 Tables). The results were also very similar after excluding former drinkers (S5 and S6 Tables).

### Influence of age, sex, APOE4, and clinical diagnosis on the association between moderate alcohol intake and Aβ positivity

As shown in Table 4, the interaction between moderate lifetime alcohol intake and age was significant, indicating that age moderates the association between moderate lifetime alcohol intake and Aβ positivity, while any interaction between moderate alcohol intake and each of sex, APOE4, and clinical diagnosis was not significant. Further subgroup analysis showed that moderate alcohol intake was significantly associated with Aβ positivity only in the older subgroup, not in the younger one (S7 Table). For the purpose of exploration, we also performed the same analyses for the moderating effect of age, sex, APOE4, and clinical diagnosis on the association between alcohol intake of <1 SD/week or 14+ SDs/week and Aβ positivity. Interaction between these 2 alcohol intake categories and each of age, sex, APOE4, and clinical diagnosis was not significant (S8 Table).

**Table 4:** Moderating effects of age, sex, APOE4, and clinical diagnosis on the association between moderate lifetime alcohol intake and amyloid-beta positivity.

Variable or interaction	OR (95% CI)^†^	*p-*Value
Model for age effect		
1–13 SDs/week	0.459 (0.211 to 1.000)	0.050
Age^a^	2.319 (1.243 to 4.325)	0.008
1–13 SDs/week × age	0.258 (0.073 to 0.918)	0.036
Model for sex effect		
1–13 SDs/week	0.253 (0.084 to 0.761)	0.014
Sex	1.716 (0.791 to 3.726)	0.172
1–13 SDs/week × sex	1.158 (0.285 to 4.706)	0.838
Model for APOE4 effect		
1–13 SDs/week	0.310 (0.137 to 0.702)	0.005
APOE4	6.397 (3.193 to 12.816)	<0.001
1–13 SDs/week × APOE4	0.898 (0.268 to 3.011)	0.862
Model for clinical diagnosis effect		
1–13 SDs/week	0.349 (0.144 to 0.845)	0.020
Clinical diagnosis^b^	5.088 (2.577 to 10.047)	<0.001
1–13 SDs/week × clinical diagnosis	0.817 (0.242 to 2.761)	0.745

^†^By multiple logistic regression analysis controlling for age, sex, APOE4, vascular risk score, and Geriatric Depression Scale score as covariates when appropriate.

^a^Younger (<75 years) versus older (≥75 years).

^b^Cognitively normal versus mild cognitive impairment.

APOE4, apolipoprotein ε4; OR, odds ratio; SD, standard drink.

## Discussion

In this study, we observed that lifetime alcohol intake of 1–13 SDs/week (moderate drinking) was associated with lower cerebral Aβ deposition compared to the no drinking group in these middle- and old-aged individuals with neither dementia nor alcohol-related disorders.

The present finding of an association between moderate alcohol intake and lower Aβ deposition is in line with results from previous studies using animal or cultured cell models, which indicated that moderate alcohol intake exerts a protective effect via attenuating Aβ accumulation [17,20]. Many clinical and epidemiological studies have reported an inverse association between moderate alcohol intake and the risk of AD dementia [3,9–13], and the present findings regarding the association between moderate alcohol intake and decreased cerebral Aβ positivity may explain this inverse association.

While moderate lifetime alcohol intake had a significant association with Aβ deposition, moderate current intake did not. This difference indicates that the protective effects of moderate alcohol intake against Aβ pathology involve the chronic effects associated with long-term exposure rather than an acute effect. The significant finding for lifetime intake only also suggests that the protective association for moderate alcohol intake is not due to the inclusion of forced abstainers, i.e., those who stopped using alcohol owing to other health concerns related to problem drinking, among the reference group (i.e., non-drinkers). Forced abstainers were classified as drinkers for lifetime alcohol intake status, whereas they were classified as non-drinkers for current alcohol intake status.

Unlike for Aβ deposition, there were no associations between moderate alcohol intake and neurodegeneration or WMHs. Similarly, previous human MRI studies did not observe an association between moderate alcohol intake and cerebral gray matter volume [29] or total brain volume [30]. However, several preclinical and human studies reported that moderate alcohol intake has protective effects against vascular changes and atrophy in the brain. Studies using cultured cell or animal models showed that moderate alcohol intake is protective against ischemic brain injury [24,27], and human MRI studies have suggested that moderate alcohol intake is protective against damage to cerebral gray [23] and white [21,22] matter. These discrepancies may be related to methodological differences between studies. However, as suggested in a systematic review of the chronic effects of moderate alcohol intake on the structural and functional properties of the brain [53], the present findings based on both structural MRI (cortical thickness and WMHs) and FDG PET (cerebral glucose metabolism) measures support that moderate alcohol intake did not exert its protective effects directly through neurodegenerative or vascular mechanisms.

Although excessive alcohol intake has been related with an increased risk of cognitive decline [1–4], and U- or J-shaped association has been implied together with the decreased risk of cognitive impairment with moderate alcohol intake [3,5–16], we did not find any association between higher alcohol intake and increased AD pathologies. Alcohol-related brain damage (ARBD) [54] was suggested as an umbrella term for conditions including Wernicke–Korsakoff syndrome, alcohol-related dementia, and other forms of persistent alcohol-related cognitive impairment. ARBD encompasses a range of clinical presentations that manifest as impairments in memory, executive functioning, and judgement, which are related to frontal brain function. Several brain imaging studies also reported damage of the frontal lobe in individuals with alcoholism [55], while AD-CT and AD-CM measures mainly include temporo-parietal degeneration. Therefore, we additionally analyzed the association between alcohol intake and frontal lobe state (i.e., glucose metabolism, cortical thickness, and WMH volume of the frontal region) in order to find out if there was any ARBD-like damage with alcohol intake. As shown in S9 Table, however, we did not find any significant results from those analyses. These null findings may be because individuals with alcohol-related disorders were excluded and, as a result, heavy drinkers (14+ SDs/week) in the present study consisted of individuals without alcoholism or other severe alcohol problems.

The investigation of the influence of age on the association between moderate alcohol intake and Aβ positivity revealed that the protective effect of moderate alcohol intake on Aβ positivity was more prominent in older individuals (≥75 years) than younger ones. This finding may be due in part to age-associated increases in the Aβ positivity rate in individuals without dementia [56]. In the present study, the Aβ positivity rate was 24.5% (*n* = 62) in the younger age group and 37.3% (*n* = 57) in the older group. The relatively small proportion of Aβ+ individuals in the younger group might decrease the likelihood of detecting a significant difference. It is also possible, as mentioned above, that these age-related differences are related to the chronic effects associated with long-term alcohol exposure. In contrast, sex, APOE4, and clinical diagnosis did not have any moderating effect on the association between moderate alcohol intake and Aβ positivity.

The present study had a couple of strengths. To the best of our knowledge, this study is the first to show the association of moderate alcohol intake with Aβ accumulation in the living human brain. The study included a relatively large number of participants who were well-characterized through comprehensive clinical assessments including systematic interview for detailed alcohol drinking history and multimodal brain imaging for in vivo AD pathologies and WMHs. In addition, various potential confounders were systematically evaluated and controlled in the statistical models in order to reveal the association between alcohol intake and brain pathologies as clearly as possible. Even after controlling for all potential confounders, the findings did not change. The results were also confirmed by sensitivity analyses conducted after excluding binge or former drinkers.

Nevertheless, the present study also had several limitations that should be considered. First, because this was a cross-sectional study, causal relationships cannot be inferred from the findings. Second, in terms of lifetime alcohol intake, underestimation of drinking or retrospective recall bias may have affected the results in older individuals. However, it is unlikely that underestimation of alcohol intake was significant because harmful drinkers and individuals with a history of alcohol use disorder were excluded from the analyses, and moderate drinkers have no reason to underestimate their alcohol intake. Moreover, to reduce recall bias, information was obtained from reliable informants as well as the study participants. Additionally, a review of self-report bias in the assessment of alcohol intake suggested that this recall bias is not greater in older individuals than in the general population [57]. Third, about one-third of the study participants were diagnosed with MCI, which may also raise some concern about the accuracy of self-report for alcohol intake. However, although individuals with MCI have some problems with their recent memory, their remote memory is very well preserved [58]: It is not likely that individuals with MCI reported their history for alcohol intake more erroneously, because the self-report for lifetime alcohol intake mainly depends on remote memory rather than recent memory. In addition, even when we controlled for clinical diagnosis (CN versus MCI) as an additional covariate in Model 3 (Tables 2, 3, and S2–S7), the results were still very similar. Fourth, there are quite different alcohol intake patterns regarding the frequency and regularity of intake, and the amount of alcohol consumed in a single session, within the moderate drinking category. Although we obtained similar findings after excluding binge drinkers in sensitivity analyses, a more detailed understanding of the influence of drinking patterns is needed. Finally, although we did not find any significant association between alcohol intake and neurodegeneration or WMHs, the lack of association may reflect a lack of statistical power given the sample size.

Although further long-term follow-up investigations in larger populations with heterogeneous alcohol intake patterns are still needed, the association of moderate alcohol intake with reduced risk of pathological Aβ deposition (about one-third of the risk for no drinking) observed in the present study may suggest that moderate lifetime alcohol intake may be beneficial in preventing AD dementia or related cognitive decline.

In conclusion, the present findings from middle- and old-aged individuals with neither dementia nor alcohol-related disorders suggest that moderate lifetime alcohol intake may have some beneficial influence on AD by reducing pathological amyloid deposition rather than amyloid-independent neurodegeneration or cerebrovascular injury.

## Supporting information

S1 STROBE Checklist Strengthening the reporting of observational studies in epidemiology (STROBE) checklist. (DOCX)

S1 Interview Form Assessment of alcohol intake history. (DOCX)

S1 Method Image acquisition and preprocessing. (DOCX)

S1 Table Demographic and clinical characteristics of participants by clinical diagnosis. (DOCX)

S2 Table Results of the multiple logistic regression analyses assessing the associations of stratified alcohol intake with Aβ positivity using different thresholds of Aβ positivity in participants overall. (DOCX)

S3 Table Results of the multiple logistic and linear regression analyses assessing the associations of stratified alcohol intake with Aβ deposition in participants without binge drinking. (DOCX)

S4 Table Results of the multiple linear regression analyses assessing the associations of stratified alcohol intake with AD-CM, AD-CT, and WMHs in participants without binge drinking. (DOCX)

S5 Table Results of the multiple logistic and linear regression analyses assessing the associations of stratified alcohol intake with Aβ deposition in participants without former drinking. (DOCX)

S6 Table Results of the multiple linear regression analyses assessing the associations of stratified alcohol intake with AD-CM, AD-CT, and WMHs in participants without former drinking. (DOCX)

S7 Table Results of the multiple logistic regression analyses assessing the associations between moderate lifetime alcohol intake and Aβ positivity by age subgroup. (DOCX)

S8 Table Moderating effects of age, sex, APOE4, and clinical diagnosis on the association between each of the lifetime alcohol intake categories and Aβ positivity. (DOCX)

S9 Table Results of the multiple linear regression analyses assessing the associations of stratified alcohol intake with frontal cerebral glucose metabolism, cortical thickness, and WMHs in participants overall. (DOCX)

## Abbreviations

AD: Alzheimer disease
AD-CM: Alzheimer disease–signature cerebral glucose metabolism
AD-CT: Alzheimer disease–signature cortical thickness
APOE: apolipoprotein E
APOE4: apolipoprotein ε4
ARBD: alcohol-related brain damage
Aβ: amyloid-beta
BMI: body mass index
CDR: Clinical Dementia Rating
CERAD-K: Korean version of the Consortium to Establish a Registry for Alzheimer’s Disease
CN: cognitively normal
FDG: fluorodeoxyglucose
GDS: Geriatric Depression Scale
KBASE: Korean Brain Aging Study for Early Diagnosis and Prediction of Alzheimer’s Disease
MCI: mild cognitive impairment
MCL: minimum cost of living
MRI: magnetic resonance imaging
PET: positron emission tomography
PiB: Pittsburg compound B
ROI: region of interest
SD: standard drink
SUVR: standardized uptake value ratio
WMH: white matter hyperintensity
