# Supplementary material for: Association of moderate alcohol intake with in vivo amyloid-beta deposition in human brain: A cross-sectional study
Source: PLoS Med. 2020 Feb 25;17(2):e1003022. doi: 10.1371/journal.pmed.1003022 (PMC7041799; doi:10.1371/journal.pmed.1003022)
Supplement: S1 Interview Form — (DOCX) [file pmed.1003022.s002.docx]

| **Assessment of alcohol intake history** |
| --- |

- Trained research nurses should interview both participants and their informants semi-quantitatively to determine patterns and amounts of current and lifetime alcohol intake.

| **Pattern of Alcohol**  **intake** | **Current drinking status** | 0 = No, 1 = Yes |
| --- | --- | --- |
|  | **Amount of current drinking (during the past year)** | □□.□ SD(s)/drinking day × □□.□□ drinking day/week = □□.□□ SD(s)/week |
|  | **Lifetime drinking status** | 0 = non-drinker, 1 = former-drinker, 2 = drinker |
|  | **Amount of lifetime drinking** | □□□.□ SD(s)/week × □□ year = □□□□.□□ SD(s)/week |
|  | **Age of drinking onset** | □□ years (only for 1 or 2 in lifetime drinking status) |
|  | **Age of drinking stop** | □□ years (only for 1 or 2 in lifetime drinking status) |

One standard drink (SD) is defined as any drink that contains 10 grams of pure alcohol according to the World Health Organization (WHO) guideline (Available from: <https://www.who.int/substance_abuse/publications/audit_sbi/en/>.) As there are a wide variety of alcoholic beverages and brands, the approximate number of SD in different beverages were categorized as follows. Research nurses should record by referring to the below SD conversion table.

| **1 standard drink (SD)** | | | | |
| --- | --- | --- | --- | --- |
| **Alcohol type** | **Alcohol volume**  **(ml)** | **Alcohol content (%)** | **Conversion value** | **Amount of pure alcohol (g)** |
| **1 glass of beer** | 250 | 4.5 | 0.8 | 9 |
| **1 glass of local Korean spirit** | 50-60 | 20 | 0.8 | 8-9.6 |
| **1 glass of spirit** | 30 | 40 | 0.8 | 9.6 |
| **1 glass of Korean traditional wine** | 150 | 8 | 0.8 | 9.6 |
| **1 glass of wine** | 100 | 12 | 0.8 | 9.6 |

| **SD conversion table** | | | | | |
| --- | --- | --- | --- | --- | --- |
| **1 can of beer**  **(330ml)** | **1 bottle of beer**  **(640ml)** | **1 bottle of local Korean spirit**  **(360ml)** | **1 bottle of spirit**  **(750ml)** | **1 bottle of Korean traditional wine**  **(900ml)** | **1 bottle of wine**  **(900ml)** |
| 1 SD | 2 SDs | 6 SDs | 24 SDs | 6 SDs | 9 SDs |

|  |  |  |  |
| --- | --- | --- | --- |
