# Supplementary material for: Association of moderate alcohol intake with in vivo amyloid-beta deposition in human brain: A cross-sectional study
Source: PLoS Med. 2020 Feb 25;17(2):e1003022. doi: 10.1371/journal.pmed.1003022 (PMC7041799; doi:10.1371/journal.pmed.1003022)
Supplement: S1 Method — (DOCX) [file pmed.1003022.s003.docx]

**S1 Method.** **Image acquisition and preprocessing**

**[^11^C]** **Pittsburg compound B (PiB) – positron emission tomography (PET) image acquisition and preprocessing**

Participants underwent simultaneous three-dimensional (3D) PiB-PET and 3D T1-weighted magnetic resonance (MR) imaging using a 3.0T Biograph mMR scanner (PET-MR scanner; Siemens, Washington DC, USA) according to the manufacturer’s approved guidelines. After intravenous administration of 555 MBq of [^11^C] PiB (range, 450-610 MBq), a 30-minute emission scan was obtained 40 minutes after injection. The PiB-PET data collected in list mode were processed for routine corrections such as uniformity, UTE-based attenuation, and decay corrections, and were reconstructed into a 256 x 256 image matrix using iterative methods (6 iterations with 21 subsets). The following image preprocessing steps were performed using Statistical Parametric Mapping 12 (SPM12; http://www.fil.ion.ucl.ac.uk/spm12) implemented in Matlab 2015b (Mathworks, Natick, MA, USA). Static PiB-PET images were co-registered to individual T1 structural images and transformation parameters for the spatial normalization of individual T1 images to a standard Montreal Neurological Institute (MNI) template were calculated. Using IBASPM software, we used the inverse transformation parameters to transform coordinates from the automatic anatomic labeling (AAL) 116 atlas [1] into an individual space for each subject (a resampling voxel size = 1 x 0.98 x 0.98 mm), and the non-gray matter portions of the atlas were individually masked using the cerebral gray matter segment image from each subject. The mean regional ^11^C -PiB uptake values from cerebral regions were extracted using the individual AAL116 atlas from T1-coregistered PiB-PET images. Cerebellar gray matter was used as the reference region for quantitative normalization of cerebral PiB uptake values due to its relatively low Aβ deposition [2]. To measure PiB uptake in the cerebellar gray matter regions, a probabilistic cerebellar atlas (Institute of Cognitive Neuroscience, UCL; Cognitive Neuroscience Laboratory, Royal Holloway) was transformed into individual space in the same manner as described above. Of the 28 anatomical structural regions in the cerebellar atlas, all cerebellar lobular regions except the vermis were included to extract the mean cerebellar uptake values.

**[^18^F] Fluorodeoxyglucose (FDG)-PET image acquisition and preprocessing**

The participants fasted for at least 6 hours and rested in a waiting room for 40 minutes prior to the scans after intravenous administration of 0.1 mCi/Kg of [^18^F] FDG radioligands. The PET data collected in list mode (5 minutes x 4 frames) were processed for routine corrections such as uniformity, UTE-based attenuation, and decay corrections. After inspecting the data for any significant head movements, we reconstructed them into a 20-minute summed image using iterative methods (6 iterations with 21 subsets). The following image processing steps were performed using SPM12 (http://www.fil.ion.ucl.ac.uk/spm12) implemented in Matlab 2015b (Mathworks, Natick, MA, USA). First, static FDG-PET images were co-registered to individual T1 structural images, and transformation parameters for the spatial normalization of individual T1 images to a standard MNI template were calculated and used to spatially normalize the PET images to the MNI template. After smoothing the spatially normalized FDG-PET images with a 12-mm Gaussian filter, intensity normalization was performed using the pons as the reference region. The prefrontal mask was created based on the AAL atlas [1], which included the superior frontal gyrus-dorsolateral (Frontal_Sup), superior frontal gyrus-orbital part (Frontal_Sup_Orb), middle frontal gyrus (Frontal_Mid), middle frontal gyrus-orbital part (Frontal_Mid_Orb), superior frontal gyrus-medial (Frontal_Sub_Medial), superior frontal gyrus-medial orbital (Frontal_Med_Orb), inferior frontal gyrus-opercular part (Frontal_Inf_Oper), inferior frontal gyrus-triangular part (Frontal_Inf_Tri), inferior frontal gyrus-orbital part (Frontal_Inf_Orb), and olfactory cortex of the both hemispheres. The prefrontal mask was then used to extract the regional mean of glucose metabolism in the prefrontal region of interest (Prefrontal ROI) using the REX toolbox (http://web.mit.edu/swg/software.htm).

**MR image acquisition and preprocessing**

All T1-weighted images were acquired in the sagittal orientation using the abovementioned 3.0T PET-MR machine. MR image acquisition parameters were as follows: repetition time = 1670 ms, echo time = 1.89 ms, field of view 250 mm, and 256 X 256 matrix with 1.0-mm slice thickness.

**T1 weight MRI preprocessing**

All MR images were automatically segmented using FreeSurfer version 5.3 (http://surfer.nmr.mgh.harvard.edu/) with manual correction of minor segmentation errors. Based on the Desikan–Killiany atlas [3], mean cortical thickness values were obtained from Alzheimer’s Disease (AD)-signature regions, including the entorhinal, inferior temporal, middle temporal, and fusiform gyrus according to a previous study [4]. Regarding the prefrontal ROI, the output from the FreeSurfer based on the Desikan-Killiany atlas was used to calculate the mean cortical thickness by taking the average of the lateral‐orbito‐frontal, medial‐orbito‐frontal, superior‐frontal, rostral‐middle‐frontal, caudal‐middle‐frontal, frontal‐pole, and pars‐orbitalis thickness of both hemispheres.

**Volume measurement of white matter hyperintensities**

We followed a validated automatic procedure published previously [5]. Briefly, the procedure consisted of 11 steps, including spatial coregistration of T1 and FLAIR images, fusing of T1 and FLAIR images, segmentation of T1, attainment of transformation parameters, deformation and obtainment of the white matter mask, obtainment of FLAIR within the white matter mask, intensity normalization of the masked FLAIR, nomination of candidate white matter hyperintensities (WMHs) with a designated threshold, creation of a junction map, and elimination of the junction. There were two modifications in the current processing procedure compared to the original study: (a) an optimal threshold of 70 was applied, as it was more suitable for our data compared to the threshold of 65 used in the original study; and, (b) given that individuals with acute cerebral infarcts were not enrolled in our sample, we did not use diffusion weighted imaging in the current automated procedure.

Using the final WMHs candidate image, WMHs volumes were extracted based on lobar ROIs in the native space of each subject. More specifically, the lobar ROIs template was adapted from a previously published minimal deformation template (MDT3) [6]. The acquired transformation parameter of each subject from the automated procedure was applied to the template to transform the lobar ROIs template into native space to be used for extracting WMHs volumes in each lobe.

References>

1. Tzourio-Mazoyer N, Landeau B, Papathanassiou D, Crivello F, Etard O, Delcroix N, et al. Automated anatomical labeling of activations in SPM using a macroscopic anatomical parcellation of the MNI MRI single-subject brain. NeuroImage. 2002;15(1):273–89.

2. Lopresti BJ, Klunk WE, Mathis CA, Hoge JA, Ziolko SK, Lu X, et al. Simplified quantification of Pittsburgh Compound B amyloid imaging PET studies: a comparative analysis. J Nucl Med. 2005;46(12):1959–72.

3. Desikan RS, Segonne F, Fischl B, Quinn BT, Dickerson BC, Blacker D, et al. An automated labeling system for subdividing the human cerebral cortex on MRI scans into gyral based regions of interest. NeuroImage. 2006;31(3):968–80.

4. Villeneuve S, Rabinovici GD, Cohn-Sheehy BI, Madison C, Ayakta N, Ghosh PM, et al. Existing Pittsburgh Compound-B positron emission tomography thresholds are too high: statistical and pathological evaluation. Brain. 2015;138(Pt 7):2020–33.

5. Tsai JZ, Peng SJ, Chen YW, Wang KW, Li CH, Wang JY, et al. Automated segmentation and quantification of white matter hyperintensities in acute ischemic stroke patients with cerebral infarction. PLoS ONE. 2014;9(8):e104011.

6. Kochunov P, Lancaster JL, Thompson P, Woods R, Mazziotta J, Hardies J, et al. Regional spatial normalization: toward an optimal target. J Comput Assist Tomogr. 2001;25(5):805–16.
