# Supplementary material for: Association of moderate alcohol intake with in vivo amyloid-beta deposition in human brain: A cross-sectional study
Source: PLoS Med. 2020 Feb 25;17(2):e1003022. doi: 10.1371/journal.pmed.1003022 (PMC7041799; doi:10.1371/journal.pmed.1003022)
Supplement: S1 Table — (DOCX) [file pmed.1003022.s004.docx]

| **S1** **Table.** Demographic and clinical characteristics of participants by clinical diagnosis | | | | |  |
| --- | --- | --- | --- | --- | --- |
| Characteristic | | CN^†^ | MCI^†^ | Overall^†^ | *p-*Value |
| *N* | | 280 | 134 | 414 |  |
| Age, years | | 69.5 (7.9) | 73.8 (6.8) | 70.9 (7.8) | <0.001 ^a^ |
| Male, *n/N (%)* | | 133/280 (47.5) | 47/134 (35.1) | 180/414 (43.5) | 0.020 ^b^ |
| APOE4 positivity, *n/N (%)* | | 52/279 (18.2) | 46/134 (34.3) | 98/413 (23.7) | 0.001 ^b^ |
| Vascular risk score | | 1.0 (1.0) | 1.1 (1.0) | 1.1 (1.0) | 0.323 ^a^ |
| GDS score | | 4.6 (4.9) | 10.2 (7.0) | 6.4 (6.2) | <0.001 ^a^ |
| MMSE | | 26.8 (2.6) | 22.5 (3.2) | 25.4 (3.5) | <0.001 ^a^ |
| Education, more than high school, *n/N (%)* | | 114/280 (40.7) | 28/134 (20.9) | 142/414 (34.3) | <0.001 ^b^ |
| Occupational complexity | |  |  |  | <0.001 ^b^ |
| None, *n/N (%)* | | 37/280 (13.2) | 38/133 (28.6) | 75/413 (18.2) |  |
| Skill level 1, *n/N (%)* | | 13/280 (4.6) | 15/133 (11.3) | 28/413 (6.8) |  |
| Skill level 2, *n/N (%)* | | 100/280 (35.7) | 37/133 (27.8) | 137/413 (33.2) |  |
| Skill level 3, *n/N (%)* | | 39/280 (13.9) | 16/133 (12.0) | 55/413 (13.3) |  |
| Skill level 4, *n/N (%)* | | 91/280 (32.5) | 27/133 (20.3) | 118/413 (28.6) |  |
| Annual income | |  |  |  | 0.115 ^b^ |
| <MCL, *n/N (%)* | | 20/280 (7.1) | 14/134 (10.4) | 34/414 (8.2) |  |
| $\geq$MCL, <2$\times$MCL, *n/N (%)* | | 120/280 (42.9) | 67/134 (50.0) | 187/414 (45.2) |  |
| $\geq$2$\times$MCL, *n/N (%)* | | 140/280 (50.0) | 53/134 (39.6) | 193/414 (46.6) |  |
| Body weight, kg | | 61.9/280 (9.8) | 60.0/134 (9.2) | 61.3/414 (9.6) | 0.061 ^a^ |
| BMI, kg/m^2^ | | 24.2/280 (3.0) | 24.7/134 (3.1) | 24.3/414 (3.0) | 0.101 ^a^ |
| Alcohol intake | |  |  |  |  |
| Drinking amount, lifetime | |  |  |  |  |
| Overall SD/week | | 5.5 (14.2) | 5.9 (17.0) | 5.6 (15.2) | 0.801 ^a^ |
| Categorized SD/week | |  |  |  | 0.070 ^b^ |
| 0 SD/week, *n/N (%)* | | 142/280 (50.7) | 85/134 (63.4) | 227/414 (54.8) |  |
| <1 SD/week, *n/N (%)* | | 12/280 (4.3) | 4/134 (3.0) | 16/414 (3.9) |  |
| 1–13 SDs/week, *n/N (%)* | | 95/280 (33.9) | 30/134 (22.4) | 125/414 (30.2) |  |
| 14+ SDs/week, *n/N (%)* | | 31/280 (11.1) | 15/134 (11.2) | 46/414 (11.1) |  |
| Drinking amount, current | |  |  |  |  |
| Overall SD/week | | 4.1 (12.0) | 1.7 (5.7) | 3.3 (10.5) | 0.007 ^a^ |
| Categorized SD/week | |  |  |  | 0.052 ^b^ |
| 0 SD/week, *n/N (%)* | | 176/280 (62.9) | 102/134 (76.1) | 278/414 (67.1) |  |
| <1 SD/week, *n/N (%)* | | 15/280 (5.4) | 5/134 (3.7) | 20/414 (4.8) |  |
| 1–13 SDs/week, *n/N (%)* | | 63/280 (22.5) | 21/134 (15.7) | 84/414 (20.3) |  |
| 14+ SDs/week, *n/N (%)* | | 26/280 (9.3) | 6/134 (4.5) | 32/414 (7.7) |  |
| Binge drinkers, *n/N (%)* | | 17/280 (6.1) | 5/134 (3.7) | 22/414 (5.3) | 0.361 ^b^ |
| Former drinkers, *n/N (%)* | | 38/280 (13.6) | 20/134 (14.9) | 58/414 (14.0) | 0.763 ^b^ |
| Cerebral Aβ deposition | |  |  |  |  |
| global Aβ retention, SUVR | | 1.19 (0.2) | 1.51 (0.5) | 1.29 (0.4) | <0.001 ^a^ |
| Aβ positivity, *n/N (%)* | | 50/277 (18.1) | 69/129 (53.5) | 119/406 (29.3) | <0.001 ^b^ |
| Neurodegeneration | |  |  |  |  |
| AD-CM, SUVR | | 1.42 (0.1) | 1.34 (0.1) | 1.39 (0.1) | <0.001 ^a^ |
| AD-CT, mm | | 2.86 (0.2) | 2.67 (0.3) | 2.80 (0.2) | <0.001 ^a^ |
| WMH volume, cm^3^ | | 5.70 (5.4) | 6.55 (5.5) | 5.96 (5.4) | 0.166 ^a^ |
| ^†^ Data are expressed as mean (standard deviation), unless otherwise indicated.  ^a^ By student t-test.  ^b^ By chi-square test.  Abbreviations: n/N, number of cases/ total number for each category; APOE4, apolipoprotein ε4; GDS, Geriatric Depression Scale; CN, cognitively normal; MMSE, Mini-Mental State Examination; MCL, minimum cost of living; BMI, body mass index; SD, standard drink; Aβ, amyloid-beta; AD-CM, Alzheimer’s disease-signature cerebral glucose metabolism; AD-CT, Alzheimer’s disease-signature cortical thickness; SUVR, standardized uptake value ratio; WMH, white matter hyperintensity. | | | | | |
