# Supplementary material for: Association of moderate alcohol intake with in vivo amyloid-beta deposition in human brain: A cross-sectional study
Source: PLoS Med. 2020 Feb 25;17(2):e1003022. doi: 10.1371/journal.pmed.1003022 (PMC7041799; doi:10.1371/journal.pmed.1003022)
Supplement: S2 Table — (DOCX) [file pmed.1003022.s005.docx]

| **S2 Table.** Results of the multiple logistic regression analyses assessing the associations of stratified alcohol intake with Aβ positivity using different thresholds of Aβ positivity in participants overall | | |
| --- | --- | --- |
| Alcohol intake, lifetime | OR (95% CI) ^†^, *p-*Value | |
|  | Threshold of 1.19 | Threshold of 1.40 |
| Model 1 ^a^ |  |  |
| <1 SD/week | 0.727 (0.244 to 2.166), 0.567 | 0.714 (0.196 to 2.599), 0.609 |
| 1–13 SDs/week | 0.460 (0.277 to 0.763), 0.003 | 0.612 (0.347 to 1.082), 0.091 |
| 14+ SDs/week | 0.700 (0.353 to 1.387), 0.307 | 0.651 (0.286 to 1.481), 0.306 |
| Model 2 ^b^ |  |  |
| <1 SD/week | 1.298 (0.400 to 4.208), 0.664 | 1.177 (0.293 to 4.723), 0.818 |
| 1–13 SDs/week | 0.431 (0.222 to 0.836), 0.013 | 0.399 (0.186 to 0.854), 0.018 |
| 14+ SDs/week | 0.564 (0.226 to 1.403), 0.218 | 0.307 (0.105 to 0.902), 0.032 |
| Model 3 ^c^ |  |  |
| <1 SD/week | 1.029 (0.310 to 3.415), 0.962 | 1.064 (0.245 to 4.610), 0.934 |
| 1–13 SDs/week | 0.433 (0.215 to 0.873), 0.019 | 0.418 (0.181 to 0.963), 0.040 |
| 14+ SDs/week | 0.551 (0.210 to 1.446), 0.226 | 0.324 (0.103 to 1.023), 0.055 |
| ^†^ By multiple logistic regression analysis (no drinking served as the reference group).  ^a^ Not adjusted.  ^b^ Adjusted for age, sex, apolipoprotein ε4, vascular risk score, and Geriatric Depression Scale score.  ^c^ Adjusted for covariates in Model 2 plus education, clinical diagnosis, occupational complexity, annual income, body weight, and body mass index.  .  Abbreviations: Aβ, amyloid-beta; OR, odds ratio; CI, confidence interval; SD, standard drink. | | |
