# Supplementary material for: Association of moderate alcohol intake with in vivo amyloid-beta deposition in human brain: A cross-sectional study
Source: PLoS Med. 2020 Feb 25;17(2):e1003022. doi: 10.1371/journal.pmed.1003022 (PMC7041799; doi:10.1371/journal.pmed.1003022)
Supplement: S3 Table — (DOCX) [file pmed.1003022.s006.docx]

| **S3 Table.** Results of the multiple logistic and linear regression analyses assessing the associations of stratified alcohol intake with Aβ deposition in participants without binge drinking | | | |
| --- | --- | --- | --- |
| Alcohol intake | Aβ positivity | | Aβ retention, SUVR |
|  | OR (95% CI) ^†^, *p-*Value | B (95% CI) ^‡^, *p-*Value | |
| Lifetime |  |  | |
| Model 1 ^a^ |  |  | |
| <1 SD/week | 0.850 (0.285 to 2.535), 0.771 | 0.014 (-0.105 to 0.132), 0.821 | |
| 1–13 SDs/week | 0.442 (0.258 to 0.760), 0.003 | -0.057 (-0.110 to -0.005), 0.033 | |
| 14+ SDs/week | 0.801 (0.350 to 1.835), 0.601 | -0.052 (-0.141 to 0.037), 0.249 | |
| Model 2 ^b^ |  |  | |
| <1 SD/week | 1.425 (0.436 to 4.656), 0.558 | 0.055 (-0.056 to 0.166), 0.329 | |
| 1–13 SDs/week | 0.328 (0.161 to 0.669), 0.002 | -0.066 (-0.124 to -0.008), 0.025 | |
| 14+ SDs/week | 0.419 (0.142 to 1.233), 0.114 | -0.099 (-0.192 to -0.006), 0.036 | |
| Model 3 ^c^ |  |  | |
| <1 SD/week | 1.260 (0.375 to 4.234), 0.709 | 0.034 (-0.070 to 0.138), 0.524 | |
| 1–13 SDs/week | 0.340 (0.161 to 0.717), 0.005 | -0.051 (-0.105 to 0.004), 0.068 | |
| 14+ SDs/week | 0.429 (0.141 to 1.303), 0.135 | -0.095 (-0.182 to -0.007), 0.034 | |
|  |  |  | |
| Current |  |  | |
| Model 1 ^a^ |  |  | |
| <1 SD/week | 0.872 (0.324 to 2.344), 0.785 | 0.009 (-0.097 to 0.115), 0.864 | |
| 1–13 SDs/week | 0.484 (0.261 to 0.898), 0.021 | -0.062 (-0.121 to -0.003), 0.039 | |
| 14+ SDs/week | 0.555 (0.151 to 2.038), 0.375 | -0.060 (-0.186 to 0.065), 0.343 | |
| Model 2 ^b^ |  |  | |
| <1 SD/week | 1.146 (0.381 to 3.443), 0.808 | 0.031 (-0.068 to 0.130), 0.539 | |
| 1–13 SDs/week | 0.496 (0.243 to 1.015), 0.055 | -0.046 (-0.103 to 0.012), 0.117 | |
| 14+ SDs/week | 0.824 (0.188 to 3.618), 0.797 | -0.025 (-0.147 to 0.097), 0.688 | |
| Model 3 ^c^ |  |  | |
| <1 SD/week | 1.193 (0.388 to 3.669), 0.758 | 0.027 (-0.065 to 0.119), 0.563 | |
| 1–13 SDs/week | 0.507 (0.240 to 1.073), 0.076 | -0.036 (-0.090 to 0.018), 0.189 | |
| 14+ SDs/week | 0.971 (0.213 to 4.433), 0.970 | -0.019 (-0.133 to 0.094), 0.742 | |
| ^†^ By multiple logistic regression analysis (no drinking served as the reference group).  ^‡^ By multiple linear regression analysis (no drinking served as the reference group).  ^a^ Not adjusted.  ^b^ Adjusted for age, sex, apolipoprotein ε4, vascular risk score, and Geriatric Depression Scale score.  ^c^ Adjusted for covariates in Model 2 plus education, clinical diagnosis, occupational complexity, annual income, body weight, and body mass index.  Global Aβ retention was used after natural log-transformation to achieve normal distribution.  Abbreviations: Aβ, amyloid-beta; SUVR, standardized uptake value ratio; OR, odds ratio; B, unstandardized regression coefficient; CI, confidence interval; SD, standard drink. | | | |
