# Supplementary material for: Association of moderate alcohol intake with in vivo amyloid-beta deposition in human brain: A cross-sectional study
Source: PLoS Med. 2020 Feb 25;17(2):e1003022. doi: 10.1371/journal.pmed.1003022 (PMC7041799; doi:10.1371/journal.pmed.1003022)
Supplement: S4 Table — (DOCX) [file pmed.1003022.s007.docx]

| **S4 Table.** Results of the multiple linear regression analyses assessing the associations of stratified alcohol intake with AD-CM, AD-CT, and WMHs in participants without binge drinking | | | |
| --- | --- | --- | --- |
| Alcohol intake |  | B (95% CI) ^†^, *p-*Value |  |
|  | AD-CM, SUVR | AD-CT, mm | WMHs, cm^3^ |
| Lifetime |  |  |  |
| Model 1 ^a^ |  |  |  |
| <1 SD/week | 0.020 (-0.047 to 0.088), 0.555 | <0.001 (-0.113 to 0.114), 0.998 | 1.430 (-1.596 to 4.456), 0.353 |
| 1–13 SDs/week | 0.028 (-0.002 to 0.058), 0.064 | 0.013 (-0.038 to 0.064), 0.614 | -0.793 (-2.066 to 0.479), 0.221 |
| 14+ SDs/week | 0.006 (-0.045 to 0.057), 0.812 | -0.049 (-0.135 to 0.038), 0.270 | 0.256 (-1.912 to 2.425), 0.816 |
| Model 2 ^b^ |  |  |  |
| <1 SD/week | 0.013 (-0.055 to 0.080), 0.709 | -0.029 (-0.128 to 0.071), 0.573 | 1.575 (-1.453 to 4.603),  0.307 |
| 1–13 SDs/week | 0.026 (-0.009 to 0.061), 0.144 | 0.006 (-0.046 to 0.059), 0.812 | -1.070 (-2.563 to 0.423), 0.160 |
| 14+ SDs/week | 0.026 (-0.031 to 0.082), 0.367 | 0.008 (-0.077 to 0.093), 0.851 | -0.874 (-3.292 to 1.543), 0.477 |
| Model 3 ^c^ |  |  |  |
| <1 SD/week | 0.018 (-0.048 to 0.083), 0.593 | -0.018 (-0.111 to 0.076), 0.711 | 1.776 (-1.277 to 4.828), 0.253 |
| 1–13 SDs/week | 0.017 (-0.017 to 0.052), 0.314 | -0.007 (-0.057 to 0.042), 0.775 | -1.009 (-2.520 to 0.502), 0.190 |
| 14+ SDs/week | 0.023 (-0.032 to 0.078), 0.417 | 0.018 (-0.062 to 0.099), 0.651 | -0.688 (-3.138 to 1.762), 0.581 |
|  |  |  |  |
| Current |  |  |  |
| Model 1 ^a^ |  |  |  |
| <1 SD/week | 0.026 (-0.034 to 0.087), 0.391 | 0.015 (-0.087 to 0.117), 0.774 | 0.397 (-2.263 to 3.057), 0.769 |
| 1–13 SDs/week | 0.016 (-0.018 to 0.050), 0.353 | 0.025 (-0.032 to 0.083), 0.384 | -0.210 (-1.641 to 1.220), 0.773 |
| 14+ SDs/week | 0.053 (-0.017 to 0.122), 0.136 | 0.045 (-0.075 to 0.165), 0.465 | -1.095 (-4.113 to 1.924), 0.476 |
| Model 2 ^b^ |  |  |  |
| <1 SD/week | 0.023 (-0.037 to 0.083), 0.450 | 0.001 (-0.087 to 0.089), 0.985 | 0.566 (-2.058 to 3.190), 0.672 |
| 1–13 SDs/week | 0.009 (-0.025 to 0.044), 0.595 | 0.008 (-0.044 to 0.060), 0.763 | -0.118 (-1.593 to 1.357), 0.875 |
| 14+ SDs/week | 0.042 (-0.029 to 0.113), 0.247 | 0.020 (-0.089 to 0.128), 0.723 | -1.104 (-4.186 to 1.978), 0.482 |
| Model 3 ^c^ |  |  |  |
| <1 SD/week | 0.023 (-0.035 to 0.080), 0.434 | -0.001 (-0.084 to 0.081), 0.976 | 0.534 (-2.102 to 3.171), 0.690 |
| 1–13 SDs/week | 0.006 (-0.028 to 0.039), 0.746 | -0.003 (-0.052 to 0.046), 0.895 | -0.120 (-1.603 to 1.364), 0.874 |
| 14+ SDs/week | 0.041 (-0.028 to 0.110), 0.242 | 0.016 (-0.086 to 0.118), 0.758 | -0.991 (-4.090 to 2.108), 0.530 |
| ^†^ By multiple linear regression analysis (no drinking served as the reference group).  ^a^ Not adjusted.  ^b^ Adjusted for age, sex, apolipoprotein ε4, vascular risk score, and Geriatric Depression Scale score.  ^c^ Adjusted for covariates in Model 2 plus education, clinical diagnosis, occupational complexity, annual income, body weight, and body mass index.  Abbreviations: Aβ, amyloid-beta; AD-CM, Alzheimer’s disease-signature cerebral glucose metabolism; SUVR, standardized uptake value ratio; AD-CT, Alzheimer’s disease-signature cortical thickness; WMHs, white matter hyperintensities; B, unstandardized regression coefficient; CI, confidence interval; SD, standard drink. | | | |
