# Supplementary material for: Association of moderate alcohol intake with in vivo amyloid-beta deposition in human brain: A cross-sectional study
Source: PLoS Med. 2020 Feb 25;17(2):e1003022. doi: 10.1371/journal.pmed.1003022 (PMC7041799; doi:10.1371/journal.pmed.1003022)
Supplement: S5 Table — (DOCX) [file pmed.1003022.s008.docx]

| **S5 Table.** Results of the multiple logistic and linear regression analyses assessing the associations of stratified alcohol intake with Aβ deposition in participants without former drinking | | | |
| --- | --- | --- | --- |
| Alcohol intake | Aβ positivity | | Aβ retention, SUVR |
|  | OR (95% CI) ^†^, *p-*Value | B (95% CI) ^‡^, *p-*Value | |
| Lifetime |  |  | |
| Model 1 ^a^ |  |  | |
| <1 SD/week | 1.039 (0.336 to 3.209), 0.947 | 0.037 (-0.089 to 0.163), 0.565 | |
| 1–13 SDs/week | 0.374 (0.198 to 0.707), 0.002 | -0.077 (-0.135 to -0.018), 0.010 | |
| 14+ SDs/week | 0.488 (0.191 to 1.249), 0.135 | -0.063 (-0.153 to 0.027), 0.172 | |
| Model 2 ^b^ |  |  | |
| <1 SD/week | 2.093 (0.612 to 7.152), 0.239 | 0.089 (-0.029 to 0.208), 0.140 | |
| 1–13 SDs/week | 0.274 (0.121 to 0.618), 0.002 | -0.073 (-0.135 to -0.011), 0.021 | |
| 14+ SDs/week | 0.386 (0.117 to 1.273), 0.118 | -0.063 (-0.161 to 0.035), 0.205 | |
| Model 3 ^c^ |  |  | |
| <1 SD/week | 1.942 (0.546 to 6.905), 0.305 | 0.070 (-0.040 to 0.179), 0.214 | |
| 1–13 SDs/week | 0.295 (0.125 to 0.699), 0.006 | -0.052 (-0.110 to 0.005), 0.075 | |
| 14+ SDs/week | 0.415 (0.119 to 1.443), 0.166 | -0.048 (-0.138 to 0.043), 0.301 | |
|  |  |  | |
| Current |  |  | |
| Model 1 ^a^ |  |  | |
| <1 SD/week | 0.807 (0.298 to 2.184), 0.673 | 0.004 (-0.103 to 0.111), 0.943 | |
| 1–13 SDs/week | 0.448 (0.239 to 0.840), 0.012 | -0.068 (-0.128 to -0.007), 0.028 | |
| 14+ SDs/week | 0.314 (0.105 to 0.937), 0.038 | -0.079 (-0.179 to 0.013), 0.093 | |
| Model 2 ^b^ |  |  | |
| <1 SD/week | 0.935 (0.299 to 2.925), 0.908 | 0.021 (-0.081 to 0.123), 0.688 | |
| 1–13 SDs/week | 0.359 (0.162 to 0.797), 0.012 | -0.060 (-0.123 to 0.004), 0.064 | |
| 14+ SDs/week | 0.400 (0.113 to 1.420), 0.156 | -0.046 (-0.146 to 0.053), 0.363 | |
| Model 3 ^c^ |  |  | |
| <1 SD/week | 0.976 (0.300 to 3.182), 0.968 | 0.020 (-0.074 to 0.114), 0.676 | |
| 1–13 SDs/week | 0.376 (0.162 to 0.875), 0.023 | -0.044 (-0.103 to 0.014), 0.139 | |
| 14+ SDs/week | 0.434 (0.115 to 1.639), 0.219 | -0.033 (-0.125 to 0.059), 0.484 | |
| ^†^ By multiple logistic regression analysis (no drinking served as the reference group).  ^‡^ By multiple linear regression analysis (no drinking served as the reference group).  ^a^ Not adjusted.  ^b^ Adjusted for age, sex, apolipoprotein ε4, vascular risk score, and Geriatric Depression Scale score.  ^c^ Adjusted for covariates in Model 2 plus education, clinical diagnosis, occupational complexity, annual income, body weight, and body mass index.  Global Aβ retention was used after natural log-transformation to achieve normal distribution.  Abbreviations: Aβ,amyloid-beta; SUVR, standardized uptake value ratio; OR, odds ratio; B, unstandardized regression coefficient; CI, confidence interval; SD, standard drink. | | | |
