# Supplementary material for: Association of moderate alcohol intake with in vivo amyloid-beta deposition in human brain: A cross-sectional study
Source: PLoS Med. 2020 Feb 25;17(2):e1003022. doi: 10.1371/journal.pmed.1003022 (PMC7041799; doi:10.1371/journal.pmed.1003022)
Supplement: S6 Table — (DOCX) [file pmed.1003022.s009.docx]

| **S6 Table.** Results of the multiple linear regression analyses assessing the associations of stratified alcohol intake with AD-CM, AD-CT, and WMHs in participants without former drinking | | | |
| --- | --- | --- | --- |
| Alcohol intake |  | B (95% CI) ^†^, *p-*Value |  |
|  | AD-CM, SUVR | AD-CT, mm | WMHs, cm^3^ |
| Lifetime |  |  |  |
| Model 1 ^a^ |  |  |  |
| <1 SD/week | 0.014 (-0.057 to 0.084), 0.699 | -0.002 (-0.117 to 0.112), 0.970 | 1.634 (-1.822 to 5.090), 0.353 |
| 1–13 SDs/week | 0.038 (0.005 to 0.070), 0.024 | 0.036 (-0.018 to 0.091), 0.188 | -0.437 (-1.944 to 1.069), 0.568 |
| 14+ SDs/week | 0.003 (-0.047 to 0.054), 0.892 | 0.075 (-0.007 to 0.157), 0.073 | -0.421 (-2.748 to 1.097), 0.722 |
| Model 2 ^b^ |  |  |  |
| <1 SD/week | 0.004 (-0.066 to 0.074), 0.904 | -0.033 (-0.136 to 0.069), 0.522 | 1.941(-1.496 to 5.377),  0.267 |
| 1–13 SDs/week | 0.031 (-0.006 to 0.068), 0.099 | 0.022 (-0.032 to 0.077), 0.424 | -0.735 (-2.420 to 0.951), 0.392 |
| 14+ SDs/week | -0.003 (-0.061 to 0.055), 0.927 | 0.052 (-0.033 to 0.137), 0.225 | -0.801 (-3.453 to 1.851), 0.553 |
| Model 3 ^c^ |  |  |  |
| <1 SD/week | 0.008 (-0.060 to 0.076), 0.815 | -0.028 (-0.125 to 0.068), 0.565 | 2.036 (-1.438 to 5.511), 0.250 |
| 1–13 SDs/week | 0.023 (-0.011 to 0.059), 0.196 | 0.009 (-0.043 to 0.061), 0.735 | -0.720 (-2.425 to 0.984), 0.406 |
| 14+ SDs/week | -0.006 (-0.062 to 0.050), 0.824 | 0.045 (-0.035 to 0.126), 0.265 | -0.730 (-3.401 to 1.940), 0.591 |
|  |  |  |  |
| Current |  |  |  |
| Model 1 ^a^ |  |  |  |
| <1 SD/week | 0.029 (-0.031 to 0.089), 0.339 | 0.008 (-0.089 to 0.104), 0.874 | 0.291 (-2.530 to 3.112), 0.839 |
| 1–13 SDs/week | 0.018 (-0.015 to 0.052), 0.284 | 0.022 (-0.034 to 0.077), 0.442 | 0.010 (-1.542 to 1.563), 0.989 |
| 14+ SDs/week | 0.043 (-0.008 to 0.093), 0.095 | 0.121 (0.038 to 0.204), 0.004 | -1.226 (-3.555 to 1.102), 0.301 |
| Model 2 ^b^ |  |  |  |
| <1 SD/week | 0.027 (-0.033 to 0.087), 0.377 | <0.001 (-0.087 to 0.087), 0.998 | 0.099 (-2.734 to 2.932), 0.945 |
| 1–13 SDs/week | 0.016 (-0.022 to 0.053), 0.404 | 0.009 (-0.046 to 0.065), 0.741 | -0.280 (-1.998 to 1.439), 0.749 |
| 14+ SDs/week | 0.028 (-0.030 to 0.086), 0.336 | 0.074 (-0.012 to 0.159), 0.091 | -1.532 (-4.179 to 1.116), 0.256 |
| Model 3 ^c^ |  |  |  |
| <1 SD/week | 0.025 (-0.033 to 0.083), 0.396 | -0.002 (-0.084 to 0.080), 0.954 | 0.076 (-2.777 to 2.929), 0.958 |
| 1–13 SDs/week | 0.010 (-0.027 to 0.046), 0.596 | -0.003 (-0.056 to 0.049), 0.902 | -0.230 (-1.966 to 1.507), 0.795 |
| 14+ SDs/week | 0.027 (-0.029 to 0.083), 0.350 | 0.072 (-0.009 to 0.152), 0.080 | -1.481 (-4.152 to 1.190), 0.276 |
| ^†^ By multiple linear regression analysis (no drinking served as the reference group).  ^a^ Not adjusted.  ^b^ Adjusted for age, sex, apolipoprotein ε4, vascular risk score, and Geriatric Depression Scale; score.  ^c^ Adjusted for covariates in Model 2 plus education, clinical diagnosis, occupational complexity, annual income, body weight, and body mass index.  Abbreviations: Aβ, amyloid-beta; AD-CM, Alzheimer’s disease-signature cerebral glucose metabolism; SUVR, standardized uptake value ratio; AD-CT, Alzheimer’s disease-signature cortical thickness; WMHs, white matter hyperintensities; B, unstandardized regression coefficient; CI, confidence interval; SD, standard drink. | | | |
