# Supplementary material for: Association of moderate alcohol intake with in vivo amyloid-beta deposition in human brain: A cross-sectional study
Source: PLoS Med. 2020 Feb 25;17(2):e1003022. doi: 10.1371/journal.pmed.1003022 (PMC7041799; doi:10.1371/journal.pmed.1003022)
Supplement: S7 Table — (DOCX) [file pmed.1003022.s010.docx]

| **S7 Table.** Results of the multiple logistic regression analyses assessing the associations between moderate lifetime alcohol intake and Aβ positivity by age subgroup | | |
| --- | --- | --- |
|  | OR (95% CI) ^†^ | *p-*Value |
| Age |  |  |
| Younger (<75 y) (n = 258) |  |  |
| <1 SD/week | 1.567 (0.352 to 6.982) | 0.556 |
| 1–13 SDs/week | 0.549 (0.243 to 1.243) | 0.151 |
| 14+ SDs/week | 0.359 (0.099 to 1.302) | 0.119 |
| Older (≥75 y) (n = 156) |  |  |
| <1 SD/week | 0.905 (0.117 to 7.028) | 0.924 |
| 1–13 SDs/week | 0.080 (0.021 to 0.299) | <0.001 |
| 14+ SDs/week | 0.257 (0.058 to 1.129) | 0.072 |
| ^†^ By multiple logistic regression analysis controlling for sex, apolipoprotein ε, vascular risk score, and Geriatric Depression Scale score (no drinking served as the reference group).  Abbreviations: Aβ, amyloid-beta; OR, odds ratio; CI, confidence interval; SD, standard drink. | | |
