# Supplementary material for: Association of moderate alcohol intake with in vivo amyloid-beta deposition in human brain: A cross-sectional study
Source: PLoS Med. 2020 Feb 25;17(2):e1003022. doi: 10.1371/journal.pmed.1003022 (PMC7041799; doi:10.1371/journal.pmed.1003022)
Supplement: S8 Table — (DOCX) [file pmed.1003022.s011.docx]

| **S8 Table.** Moderating effects of age, sex, APOE4, and clinical diagnosis on the association between each of the lifetime alcohol intake categories and Aβ positivity | | |
| --- | --- | --- |
|  | OR (95% CI) ^†^ | *p-*Value |
| Model for age effect |  |  |
| Lifetime alcohol intake |  |  |
| <1 SD/week | 1.454 (0.343 to 6.168) | 0.612 |
| 1–13 SDs/week | 0.459 (0.211 to 1.000) | 0.050 |
| 14+ SDs/week | 0.299 (0.090 to 0.987) | 0.048 |
| Age ^a^ | 2.319 (1.243 to 4.325) | 0.008 |
| Lifetime alcohol intake$\times$Age |  |  |
| <1 SD/week$\times$Age | 0.905 (0.087 to 9.462) | 0.934 |
| 1–13 SDs/week$\times$Age | 0.258 (0.073 to 0.918) | 0.036 |
| 14+ SDs/week$\times$Age | 1.268 (0.253 to 6.367) | 0.773 |
| Model for sex effect |  |  |
| Lifetime alcohol intake |  |  |
| <1 SD/week | 1.189 (0.219 to 6.468) | 0.841 |
| 1–13 SDs/week | 0.253 (0.084 to 0.761) | 0.014 |
| 14+ SDs/week | 3.776 (0.266 to 53.654) | 0.326 |
| Sex | 1.716 (0.791 to 3.726) | 0.172 |
| Lifetime alcohol intake$\times$sex |  |  |
| <1 SD/week$\times$sex | 1.260 (0.123 to 12.860) | 0.846 |
| 1–13 SDs/week$\times$sex | 1.158 (0.285 to 4.706) | 0.838 |
| 14+ SDs/week$\times$sex | 0.070 (0.004 to 1.226) | 0.069 |
| Model for APOE4 effect |  |  |
| Lifetime alcohol intake |  |  |
| <1 SD/week | 1.681 (0.518 to 5.457) | 0.387 |
| 1–13 SDs/week | 0.310 (0.137 to 0.702) | 0.005 |
| 14+ SDs/week | 0.380 (0.122 to 1.187) | 0.096 |
| APOE4 | 6.397 (3.193 to 12.816) | <0.001 |
| Lifetime alcohol intake$\times$APOE4 |  |  |
| <1 SD/week$\times$APOE4 | NA | NA |
| 1–13 SDs/week$\times$APOE4 | 0.898 (0.268 to 3.011) | 0.862 |
| 14+ SDs/week$\times$APOE4 | 0.814 (0.165 to 4.020) | 0.800 |
| Model for clinical diagnosis effect |  |  |
| Lifetime alcohol intake |  |  |
| <1 SD/week | 1.383 (0.332 to 5.752) | 0.656 |
| 1–13 SDs/week | 0.349 (0.144 to 0.845) | 0.020 |
| 14+ SDs/week | 0.449 (0.138 to 1.454) | 0.182 |
| Clinical diagnosis ^b^ | 5.088 (2.577 to 10.047) | <0.001 |
| Lifetime alcohol intake$\times$clinical diagnosis |  |  |
| <1 SD/week$\times$clinical diagnosis | 0.772 (0.064 to 9.350) | 0.839 |
| 1–13 SDs/week$\times clinical diagnosis$ | 0.817 (0.242 to 2.761) | 0.745 |
| 14+ SDs/week$\times$clinical diagnosis | 0.596 (0.120 to 2.950) | 0.525 |
| ^†^ By multiple logistic regression analysis controlling for age, sex, apolipoprotein ε4, vascular risk score, and Geriatric Depression Scale score as covariates when appropriate  Abbreviations: Aβ, amyloid-beta; OR, odds ratio; CI, confidence interval; SD, standard drink; NA, not applicable.  ^a^ Younger (< 75 years) versus older (≥ 75 years) | | |

^b^ Cognitively normal versus mild cognitive impairment
