# Supplementary material for: Association of moderate alcohol intake with in vivo amyloid-beta deposition in human brain: A cross-sectional study
Source: PLoS Med. 2020 Feb 25;17(2):e1003022. doi: 10.1371/journal.pmed.1003022 (PMC7041799; doi:10.1371/journal.pmed.1003022)
Supplement: S9 Table — (DOCX) [file pmed.1003022.s012.docx]

| **S9 Table.** Results of the multiple linear regression analyses assessing the associations of stratified alcohol intake with frontal cerebral glucose metabolism, cortical thickness, and WMHs in participants overall | | | |
| --- | --- | --- | --- |
| Alcohol intake |  | B (95% CI) ^†^, *p-*Value |  |
|  | Frontal CM, SUVR | Frontal CT, mm | Frontal WMHs, cm^3^ |
| Lifetime |  |  |  |
| Model 1 ^a^ |  |  |  |
| <1 SD/week | 0.019 (-0.048 to 0.087),  0.572 | -0.069 (-0.150 to 0.013),  0.100 | 0.308 (-1.158 to 1.774), 0.680 |
| 1–13 SDs/week | 0.014 (-0.015 to 0.044),  0.338 | -0.014 (-0.051 to 0.022),  0.438 | -0.487 (-1.094 to 0.121), 0.116 |
| 14+ SDs/week | 0.003 (-0.040 to 0.045),  0.907 | -0.059 (-0.111 to -0.007),  0.026 | -0.439 (-1.327 to 0.449), 0.332 |
| Model 2 ^b^ |  |  |  |
| <1 SD/week | 0.012 (-0.055 to 0.078),  0.727 | -0.059 (-0.135 to 0.018), 0.132 | 0.537 (-0.914 to 1.987), 0.467 |
| 1–13 SDs/week | 0.018 (-0.016 to 0.052),  0.305 | 0.020 (-0.019 to 0.060), 0.309 | -0.412 (-1.118 to 0295), 0.253 |
| 14+ SDs/week | 0.019 (-9.029 to 0.068),  0.429 | 0.004 (-0.052 to 0.060), 0.882 | -0.479 (-1.499 to 0.542), 0.357 |
| Model 3 ^c^ |  |  |  |
| <1 SD/week | 0.015 (-0.051 to 0.081),  0.652 | -0.050 (-0.125 to 0.026), 0.194 | 0.619 (-0.841 to 2.079), 0.405 |
| 1–13 SDs/week | 0.012 (-0.022 to 0.045), 0.492 | 0.021 (-0.018 to 0.060), 0.295 | -0.355 (-1.068 to 0.358), 0.328 |
| 14+ SDs/week | 0.013 (-0.035 to 0.061),  0.585 | 0.015 (-0.042 to 0.071), 0.610 | -0.380 (-1.414 to 0.654), 0.470 |
|  |  |  |  |
| Current |  |  |  |
| Model 1 ^a^ |  |  |  |
| <1 SD/week | 0.022 (-0.038 to 0.082),  0.472 | -0.056 (-0.129 to 0.018), 0.137 | -0.060 (-1.347 to 1.227), 0.927 |
| 1–13 SDs/week | 0.010 (-0.023 to 0.042), 0.566 | -0.011 (-0.051 to 0.030),  0.604 | -0.129 (-0.810 to 0.552), 0.710 |
| 14+ SDs/week | 0.038 (-0.010 to 0.087), 0.123 | -0.001 (-0.061 to 0.059),  0.979 | -0.722 (-1.746 to 0.302), 0.167 |
| Model 2 ^b^ |  |  |  |
| <1 SD/week | 0.020 (-0.038 to 0.079), 0.493 | -0.045 (-0.112 to 0.023), 0.192 | 0.146 (-1.110 to 1.403), 0.819 |
| 1–13 SDs/week | 0.008 (-0.026 to 0.041), 0.652 | 0.003 (-0.036 to 0.042), 0.887 | 0.075 (-0.621 to 0.771), 0.832 |
| 14+ SDs/week | 0.030 (-0.021 to 0.082), 0.247 | 0.023 (-0.037 to 0.083), 0.456 | -0.293 (-1.370 to 0.784), 0.593 |
| Model 3 ^c^ |  |  |  |
| <1 SD/week | 0.021 (-0.037 to 0.079), 0.472 | -0.046 (-0.113 to 0.020),  0.171 | 0.127 (-1.132 to 1.386), 0.843 |
| 1–13 SDs/week | 0.005 (-0.028 to 0.038), 0.754 | -0.001 (-0.039 to 0.038), 0.976 | 0.088 (-0.611 to 0.786), 0.805 |
| 14+ SDs/week | 0.029 (-0.022 to 0.079), 0.267 | 0.024 (-0.035 to 0.083), 0.430 | -0.296 (-1.375 to 0.784), 0.591 |
| ^†^ By multiple linear regression analysis (no drinking served as the reference group).  ^a^ Not adjusted.  ^b^ Adjusted for age, sex, apolipoprotein ε4, vascular risk score, and Geriatric Depression Scale score.  ^c^ Adjusted for covariates in Model 2 plus education, clinical diagnosis, occupational complexity, annual income, body weight, and body mass index.  Abbreviations: Aβ, amyloid-beta; SUVR, standardized uptake value ratio; WMHs, white matter hyperintensities; B, unstandardized regression coefficient; CI, confidence interval; SD, standard drink. | | | |
